# Isolated Voices: Perspectives of Teachers, School Nurses, and Administrators Regarding Implementation of Sexual Health Education Policy

**DOI:** 10.1111/josh.12853

**Published:** 2019-12-08

**Authors:** Elizabeth Dickson, Mark Parshall, Claire D. Brindis

**Affiliations:** ^1^ University of New Mexico, College of Nursing, MSC07 4380 Box 9, 1 University of New Mexico Albuquerque NM 87131; ^2^ Department of Pediatrics, Division of Adolescent Medicine, University of California San Francisco, Philip R. Lee Institute for Health Policy Studies, Adolescent and Young Adult Health National Resource Center, 3333 California Street San Francisco CA 94118

**Keywords:** sexual health education, adolescent health, secondary school, policy implementation, social ecological model

## Abstract

**BACKGROUND:**

Comprehensive sexual health education (SHE) reduces risky sexual behavior and increases protective behavior in adolescents. It is important to understand how professionals responsible for implementing SHE policy interpret state and local policy and what influences their commitment to formal SHE policy implementation.

**METHODS:**

This descriptive study explored content and delivery of SHE policy in a rural, southwestern state with high levels of poverty, unintended adolescent pregnancy, and sexually transmitted infections. The social ecological model (SEM) was used to better understand levels of influence on the implementation of SHE policy.

**RESULTS:**

We conducted telephone surveys with 38 teachers, 63 nurses, and 21 administrators in public secondary schools. There was substantial local variability in the scope and content of SHE curricula. Respondents identified significant barriers to the delivery of SHE content and minimal evaluation of whether educational objectives were met. Based on participant responses, community and organizational SEM levels had the greatest influence on SHE policy implementation, although examples of all SEM levels were identified.

**CONCLUSIONS:**

Given perceived challenges regarding subject matter, successful SHE implementation at the local level requires committed stakeholders working in concert at the school and community levels, backed by strong policy commitment at the state level.

The need for evidence‐based, medically accurate, age‐appropriate, comprehensive sexual health education (SHE) in secondary schools is well documented,^1^, ^2^, ^3^, ^4^ and multiple resources exist to support its implementation.^5^, ^6^, ^7^ SHE is identified as part of the 2020 *Healthy People* objectives family planning priority (FP‐12): “increase the proportion of adolescents who received formal instruction on reproductive health topics before they are 18 years old.”^8^ Comprehensive approaches to SHE have been identified as a preventative link between adolescent behaviors and adolescent health outcomes through strengthening protective behaviors, while reducing the incidence of risk behaviors associated with adverse health outcomes (Table 1).^11^, ^12^, ^13^, ^14^, ^15^, ^16^, ^17^

**Table 1:** Sexual Health Outcomes and Behaviors (New Mexico and United States)

	New Mexico	United States
Sexual health outcomes		
Pregnancy rate (rate per 1000 women 15‐19 years of age)*	62	43
Unintended pregnancy (percentage of women less than 20 years of age)	56%	N/A
Birth rate (rate per 1000 women 15‐19 years of age)†	43	26
STI: Chlamydia (rate per 100,000 population)‡		
Women, age 15‐24 years old	4375	3635
Men, age 15‐24 years old	1419	1327
STI: Gonorrhea (rate per 100,000 population)§		
Women, age 15‐24 years old	682	623
Men, age 15‐24 years old	536	520
Sexual behaviors (9th‐12th grade students)		
Currently sexually active^11^	27%	29%
Did not use any method to prevent pregnancy during last sexual intercourse^11^	16%	14%
Did not use condom during last sexual intercourse^11^	48%	46%

* NM ranked highest of all 50 states.^9^

† NM ranked highest of all 50 states with Arkansas and Oklahoma.

‡ NM ranked 4th among all 50 states.^10^

§ NM ranked 10th among all 50 states.^10^

State and local policies related to SHE in schools may also influence rates of unintended pregnancy and STIs among adolescents.^17^, ^18^, ^19^, ^20^ Although there is broad professional and parental support for school‐based SHE,^21^, ^22^, ^23^ extensive challenges remain at local and state levels. Given the high rates of unintended adolescent pregnancy and STIs,^9^ it is important to ascertain how state‐level SHE policy goals are being implemented in local schools and school districts.

New Mexico is a large, southwestern state with a population of 2.1 million people, 25% of whom live in rural communities.^24^ With a minority‐majority population (48% Hispanic, 11% American Indian), 36% of New Mexicans speak a language other than English at home.^24^ New Mexico has the second highest poverty rate nationally (20%),^25^ and 97% of counties contain Health Professional Shortage Areas.^24^ The state has one of the highest adolescent pregnancy rates in the United States (currently 62 per 1000 women 15‐19 years of age compared with 43 per 1000 in the United States; Table 1),^9^ the public, high school graduation rate is one of the lowest nationally (71%),^26^ and many adolescents lack access to reproductive health services.^24^ For adolescents from New Mexico communities characterized by higher risks of unintended pregnancy and STI/unplanned pregnancy and STI infections infections,^27^, ^28^, ^29^ inadequate SHE is a health equity challenge. Better understanding of prevailing modes of SHE policy implementation in public secondary schools and of the variation in what content is or is not covered would be helpful for more effective and supportive interventions.

The 3 state policies guiding SHE in New Mexico public schools are summarized in Table 2.^30^, ^31^, ^32^, ^33^ The policies outline standards for SHE content, specify student exemptions from SHE content, and affirm a health education course as a high school graduation requirement. These policies are not specific or prescriptive about content, medical accuracy, or cultural appropriateness, all important aspects for SHE‐related state policy,^34^, ^35^ nor do they require or recommend any specific curriculum. Currently, there is little publicly available data identifying who is responsible for teaching SHE and what SHE content is (or is not) being taught in the state.

**Table 2:** New Mexico State Policies Guiding Sexual Health Education

Name of Policy	Requirements
New Mexico Administrative Code (NMAC) 6.12.2.1030^30^	Local school districts must provide “instruction about HIV and related issues” in elementary through senior high school grades.
	Instruction must include “ways to reduce the risk of getting HIV/AIDS,” such as the “ability to demonstrate refusal skills, overcome peer pressure, and use decision making skills.”
New Mexico Administrative Code (NMAC) 6.29.6.9‐10^31^	Details standards and benchmarks for health education topics for students in kindergarten through 12th grade, including SHE topics.
	SHE topics mostly defined as “areas related to sexuality,” with reference to sexual behavior, contraception, condom use, sexually transmitted infection, unprotected sex, unwanted pregnancy, HIV, abstinence, etc.
	Requires districts to develop policy to allow exemption of students by their parents from any part of health education curriculum addressing sexual health (more commonly known as opt‐out).
New Mexico Statute (NMSA) 1978, Section 22‐13‐1.1.[K]^32^	Statutory requirement for high school graduation; students must complete course in health education in middle or high school; statute is not prescriptive about content.

SHE, sexual health education.

Notwithstanding legislative or regulatory directives at the state level, the depth and comprehensiveness of SHE content and how it is taught are influenced at the local level by competing curricular priorities and constraints on time and/or resources, as well as the qualifications, experience, and comfort level of individuals responsible for SHE instruction.^36^, ^37^, ^38^, ^39^, ^40^, ^41^ Local community factors (eg, strong religious or political beliefs, or perceptions of such beliefs) may also influence whether certain SHE topics are taught (eg, abortion, sexual orientation, gender identity, emergency contraception, or sexual violence).^39^, ^42^, ^43^, ^44^ They may also contribute to opposition or lack of support from parents and community members. In contrast, school staff and administrators report that clear, specific state and district policies supportive of SHE, as well as adequate resources and system capacity, can legitimize SHE within a community and reduce the pressures not to teach SHE.^37^, ^39^

The purpose of this descriptive study was to: (1) describe the content and delivery of SHE in New Mexico public secondary schools and (2) identify the levels and sources of influence on implementation of SHE policies. We asked the following research questions: (1) What SHE content do New Mexico public secondary schools offer and by whom is it delivered? and (2) What factors influence decision making about implementation of SHE policy in New Mexico public secondary schools? We surveyed public secondary school personnel to ascertain what factors contribute to the successful implementation of state policy, as well as barriers faced in fulfilling SHE policy requirements. We used the social ecological model (SEM)^45^ as a framework to organize responses to open‐ended questions regarding the levels of influence on the implementation of SHE policy. The 5 SEM levels—intrapersonal, interpersonal, organizational, community, and policy—helped to describe the nested levels of individual attributes and the social, institutional, and cultural environments that influenced policy understanding and implementation.

## METHODS

### Participants

A convenience sample of educators responsible for teaching health, school nurses, and school administrators (principals and assistant principals) who were employed in a public secondary school in New Mexico was recruited using snowball sampling from formal and informal professional networks between August 2016 and January 2017. At least one nurse, educator, and administrator was contacted from 88 of the 89 school districts in the state and, where appropriate, from both middle and high schools (not always feasible in rural communities with one school, or where employees worked at more than one school). Employees of the school districts governed by tribal authority or located on tribal land were not sampled because of practical reasons and time constraints, including separate processes and protocols to conduct research in such settings.

### Instrument

A survey tool from a similar study in California^46^ was adapted with permission for use. The original survey tool had questions on 21 topics related to SHE content. It was piloted with a small number of local school nurses, health educators, and school administrators, none of whom participated in the final study. Their feedback resulted in deletion of 2 items, addition of 3 items, and minor wording changes to 9 items to enhance relevance to local context. The survey consisted of up to 67 structured and 37 open‐ended questions covering SHE content (what is being taught), delivery (who is teaching and how), and policy understanding and implementation. Because of branching logic, not every respondent answered every structured question. Many open‐ended questions were optional follow‐ups to structured questions. Other open‐ended questions provided insight into levels of influence when implementing SHE policy or issues important to the respondent. The survey was administered via phone interviews. Participants were asked to answer questions based on their own perspectives, work experiences, and local practices.

### Procedure

Prospective participants from the 3 participant groups at secondary schools were identified through referrals, formal and informal professional networks, and/or names on public‐domain, school‐district websites. We contacted individuals by phone and/or email up to 3 times. If a participant indicated approval was needed from their school district prior to participation, we contacted the district and obtained necessary approval. Before each interview, the interviewer (a public health nurse with interview experience) read from a standardized, approved script, and verbal consent was obtained and documented. This process was repeated for each participant in each district. The interviewer entered responses directly into an encrypted, online data management platform, Research Electronic Data Capture (REDCap).^47^ Responses to open‐ended questions were read back for confirmation. After the interview, a $20 merchandise card was mailed to participants. No participant contact information and no links to school or district were maintained after completion of the interview.

### Data Analysis

Quantitative data were analyzed in SPSS Statistics for Windows, Version 23 (IBM, Armonk, NY). Descriptive statistics included frequencies and percentages, or means or medians as appropriate. Analysis of open‐ended questions involved evaluating and summarizing responses for common areas of concern or emphasis, with review and agreement among the authors. Specific, open‐ended responses were grouped in each SEM level, which were then used to evaluate different types and levels of influence on SHE policy implementation.

## RESULTS

Of 290 initial contacts, 63 school nurses, 38 educators, and 21 administrators consented to participate (N = 122, 42% response rate), representing 69% of school districts (61/88). Approximately half of the districts (51%) had more than one participant. A larger proportion of participants worked in high schools (50%) compared with middle schools (32%) or both (25%) (Table 3). Over 60% of the sample worked in counties designated as rural (22%) or mixed urban/rural (40%). Survey results are organized by content and delivery of SHE, issues surrounding SHE policy, and influences on SHE policy decisions by SEM levels of influence (Table 4).

**Table 3:** Demographics of Participants (N = 122)

		School Nurse N (%)	Educator N (%)	Administrator N (%)
Characteristic	Total N (%)	63 (52)	38 (31)	21 (17)
Type of school				
High school	50 (41)	23 (37)	19 (50)	8 (38)
Middle school	39 (32)	14 (22)	14 (37)	11 (52)
Both	30 (25)	23 (37)	5 (13)	2 (10)
Other	3 (3)	5 (5)	0	0
Urban/rural designation^48^				
Metropolitan counties	25 (21)	13 (21)	8 (21)	4 (19)
Small metropolitan counties	20 (16)	10 (16)	6 (16)	4 (19)
Mixed urban/rural counties	49 (40)	25 (40)	16 (42)	8 (38)
Rural counties	27 (22)	14 (23)	8 (21)	5 (24)

**Table 4:** Social Ecological Model Influences on Implementation of Sexual Health Education Policy: Summary of Participant Responses

SEM Level	Barriers	Facilitators
Intrapersonal‐level influences	Being responsible for deciding whether to “tone down” or avoid controversial content to avoid conflict with students, administration, parents, and community members. Feeling frustrated that policy is not clear, school administration does not support their work. Feeling unsure how to interpret what needs to be taught. Feeling alone, angry, confused, unsupported, struggling, and morally conflicted.	Confidence/comfort to teach SHE increases with training, certification, knowledge of who to contact with questions—affects how they teach students.
Interpersonal‐level influences	Conflicts with co‐workers who do not believe students need SHE.	Supportive collaboration with co‐workers helps with course and teaching content. Positive, trusting relationships with students facilitates teaching SHE content.
Organizational‐level influences	Need more than one staff member responsible for teaching content. Content is not priority since it is not tested like other core subjects. Lack of funding for training, materials, and resources makes it difficult to meet policy requirements. Need available school nursing services and/or SBHC services. Staff fear that involvement in SHE might affect their evaluation negatively, job security.	Supportive administration will schedule time for class in schedule; reduce class size; approve up‐to‐date educational content, teaching materials, training, technology; create supportive, collaborative environment for staff to work together. Require training or certification to teach SHE content. Organization demonstrates respect for local/community culture by looking to provide culturally appropriate educational materials. Supportive administrators advocate to school board and community groups for resources, trainings, outside organizational help. Having a school staff “champion” who understands and advocates for SHE in school and community, and with board.
Community‐level influences	Need for multilingual education materials. Uninterested or unsupportive school board members and parents are barriers. Political, social, and religious ideologies of community groups diminish productive policy discussions. Long travel to locations for training in rural communities.	Positive presence of an active SHAC and/or SBHC. Community individuals available as expert speakers on sexual health. Supportive community members, district leaders, and parents actively seek to understand content being taught versus making assumptions.
Policy‐level influences	State policies are often unknown by those responsible. No evaluation/review of correct policy implementation. Need for district‐specific policy addressing SHE content to support those responsible. Policy orientation needed for school staff and community members to understand policy requirements, including opt‐out policy. State policy does not clearly mandate comprehensive content. Assure availability of health care resources for students (eg, counseling, public health clinics, SBHCs).	Potential collaboration between state education and health agencies to share resources.

### Content of SHE

**Figure 1:**
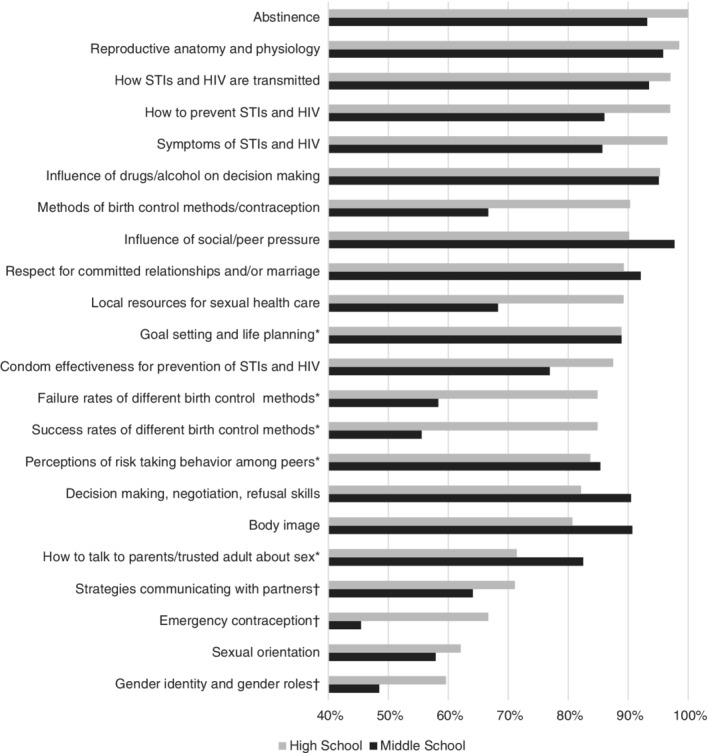
Sexual Health Education Content: Percentages of Respondents Indicating Topic Covered in High School and Middle School. *21% to 25% of Sample Replied “Do not Know” or Did Not Respond. †26% to 30% of Sample Replied “Do not Know” or Did Not Respond. STIs: Sexually Transmitted Infections.

Figure 1 summarizes the content areas participants identified as covered in middle school and high school SHE curricula. Substantially fewer middle schools than high schools covered contraception and gender identity/gender roles. By contrast, a somewhat higher percentage of middle schools than high schools covered body image; social/peer pressure; how to talk about sex with a parent or trusted adult; and decision making, negotiation, and refusal skills.

Open‐ended questions allowed participants to identify other SHE topics covered (ie, beyond those specified in the survey question response categories) and topics excluded from SHE instruction in their school(s). Eighty‐one percent of participants identified additional topics taught: sexual assault, dating violence or coercion, sexual consent, pregnancy and birth, bullying, human papillomavirus vaccination, cyber safety, and pregnancy options (including abortion). Nearly 40% of participants identified at least one topic specifically *excluded* from SHE instruction: birth control, condom use, emergency contraception, gender identity, and sexual orientation. Reasons for not covering topics included: “Our community is religious” (or “conservative”), “We don't talk about that,” “We are discouraged,” and “We are only abstinence education.”

### Delivery of SHE

**Figure 2:**
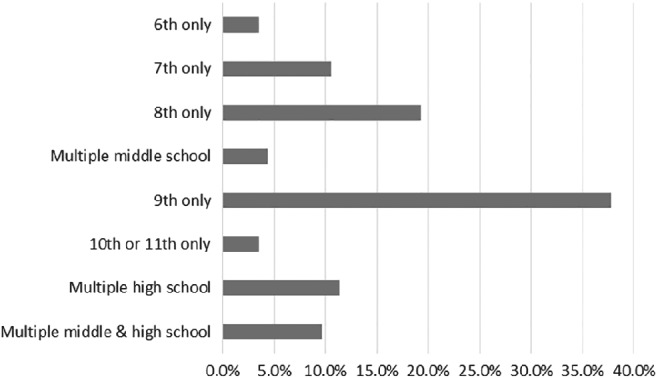
Grades Teaching Sexual Health Education Content

Approximately 87% of participants reported that SHE content was integrated as part of a required course, most commonly a health class (70%), and most commonly in eighth and ninth grades (Figure 2). Other courses included science, physical education, home economics, or family and consumer science. Almost all participants (96%) reported more than one type of instructor was involved in teaching SHE, most commonly health teachers (62%) and school nurses (34%). Approximately half of respondents indicated that delivery of SHE involved external organizations (eg, School‐Based Health Center [SBHC] staff, family planning clinics, public health department staff, medical students, local law enforcement, pregnancy crisis centers, and community groups). External speakers were valued for providing up‐to‐date information and newer teaching materials. The median (interquartile range) estimates of hours per middle and high school year devoted to SHE were 8 (3‐15) and 7 (3‐15) hours, respectively.

Approximately one‐third (34%) of participants reported additional training/certification required for teaching SHE; the other two‐thirds reported either not knowing if additional training/certification was required (30%), or that it was not (37%). Approximately half of participants reported their district was supportive of SHE training, but fewer than 10% reported attending regular training, citing administrative challenges, or limited resources as constraints.

Two‐thirds of participants reported their school(s) used textbooks to teach SHE. A similar percentage reported additional self‐developed curriculum, often because the available teaching materials were out of date. Only 15% reported using an evidence‐based curriculum, one‐third did not know whether they did, and half said they did not. Most participants (79%) believed their instructional materials were culturally appropriate, but 54% reported having only English‐language instructional materials, and 65% believed resources in other languages were needed.

### SHE Policy

Approximately 40% of the participants were familiar with school district policies for SHE or HIV/AIDS prevention education. A majority (54%) indicated that state policies governing SHE were understandable, but nonetheless criticized them for not being comprehensive or specific enough, or for not holding schools and districts accountable. Approximately 90% of participants reported not knowing whether their district or the state public education agency evaluated the delivery or outcomes of SHE.

Nearly two‐thirds of the participants (65%) reported having a district opt‐out policy that allows parents to remove their children from classes with SHE content; 10% reported an opt‐in policy, requiring active parental consent for their student to participate in SHE; and 4% reported a combination of opt‐out/opt‐in. The remaining 21% either did not know or reported their school or district did not have an opt‐out/opt‐in policy.

### Influences on Policy Implementation

Teachers, principals, school nurses, community members, and parents were acknowledged by participants as having the most influence on the delivery of SHE in their school. When asked about what made it easier or more challenging to teach SHE, participants identified barriers and facilitators to SHE policy implementation corresponding to all 5 SEM levels (Table 4).

## DISCUSSION

In this article, we describe the content and delivery of SHE in public secondary schools in a rural, southwestern state in which adolescents are at higher risk for adverse sexual health outcomes compared against national averages. Participants—educators, school nurses, and school administrators—represented front‐line school personnel responsible for SHE delivery. Barriers to teaching SHE were similar to those identified in previous studies, including insufficient training, inadequate resources, time limits to teach SHE content, political constraints, and balancing the need for SHE against other educational mandates.^37^, ^39^, ^44^, ^46^, ^49^, ^50^

### Inconsistent Implementation of SHE Policy

State legislatures often mandate health priorities in educational policy, but may be ambivalent about providing adequate administrative or financial support, such as requiring formal training as a precursor to teach SHE. As a result, state officials and district administrators are often constrained by budgetary pressures that limit time and resources devoted to SHE policy implementation,^37^, ^40^, ^44^, ^51^ often leaving the responsibility for policy implementation to personnel at the local level who may not be familiar with what the policy requires.^52^

The data regarding topics least commonly covered in both middle and high school (gender identity/roles, sexual orientation, emergency contraception, success rates of contraception) add important information to what has been reported previously as educational gaps.^43^, ^53^, ^54^, ^55^ Given the well‐documented physical and mental health risks experienced by sexual minority adolescents^56^, ^57^ and their higher risk of STIs and unintended pregnancy,^58^, ^59^ the lack of any state requirement to include content on sexual orientation and gender identity in SHE is concerning. Similarly, while a comprehensive SHE approach includes content on abstinence, abstinence‐only, or abstinence‐focused curriculum have not been shown to be effective in preparing adolescents to avoid or delay initiating sexual activity.^60^ School districts excluding content about contraception are potentially out of compliance with formal state policy.

Districts are responsible for providing curricular content that meets state requirements for graduation and have some degree of discretion in how and when that content is offered. However, approximately one‐third of respondents were not aware of state policies relevant to SHE content. This lack of awareness may be an unintended consequence of state policies that consist of broad, but vague directives. The lack of specificity may represent a state policy “compromise,” between telling local school districts what to cover and giving them considerable flexibility in interpreting and implementing state requirements in light of local community politics. This compromise may actually create more challenges for school and district staff responsible for meeting requirements without clear state guidance or sufficient resources. As a result, interpretation and implementation of state policy may result in an uneven patchwork of SHE content throughout the state that disadvantages some young people relative to others depending upon where they live.

Another policy inconsistency was reflected in some of the participants' knowledge regarding opt‐out policies. Over 20% of respondents were not familiar with or did not observe state policy mandating parents be allowed the option of removing their student from classes with SHE content. A smaller percentage reported a combination of opt‐in and opt‐out, at odds with state policy. It is critical that teachers and parents understand that parents are able to remove their student when SHE topics are taught. Understanding policy content, such as opt‐out, may in fact, assure parents of their discretion regarding their children's participation, and potentially increase support of SHE being taught in the schools.^39^

To impact and improve health outcomes, some federal funding priorities require implementation or appropriate adaptation of evidence‐based curricula.^61^, ^62^ However, only 15% of our respondents indicated they used evidence‐based SHE curriculum. It was far more common for those who taught SHE to use available textbooks or resources, even if out‐of‐date, or develop their own content, often without oversight, approval, or verification of whether it is evidence‐based. Participants might be using evidence to develop their own curriculum, but not realize it and not report the use of evidence‐based curriculum. While most respondents believed their instructional materials remained culturally appropriate, a substantial majority reported needing materials in languages other than English. However, we did not ask if there were instructors capable of teaching in languages other than English.

### Need for SHE Training

The quality of SHE instruction is affected by whether teachers have specific training.^63^ Participants identified their need for recurring training/professional development and additional resources as important reasons for relying on outside presenters to teach SHE content. Overwhelmingly, participants wanted their classes taught in compliance with required standards. However, they did not always understand the policy standards or know whether training was available. They often lacked teaching materials or resources to support building the capacity to deliver evidence‐based SHE. It is concerning over 90% of participants reported that they or others responsible for SHE were seldom able to attend trainings, due to travel distance and expense, difficulty taking time off, or lack of funding.^39^

### SEM Influences on SHE Policy Implementation

An individual's own experiences and beliefs, combined with different degrees of self‐efficacy and comfort teaching SHE content, could serve as either facilitators or barriers at the intrapersonal level, and shape the respondents' level of personal commitment to teaching SHE. The personal challenge of SHE policy implementation was clear in participant reports of feeling alone when deciding how to interpret SHE policy for their students.^39^ Thus, if they felt little confidence in teaching SHE content, this lack of external support could contribute to their avoidance. On the other hand, external support and validation of teaching SHE could overcome the individual's lack of self‐confidence. Considering implications of local interpretation of policy,^64^ these results point to a need for greater clarification, guidance, and support of SHE policy requirements and expected implementation by school districts and school staff responsible for teaching SHE.

Positive relationships between health and education staff, and sharing of respective expertise to meet the needs of students were important facilitators of SHE at the interpersonal level. However, the ability to reconcile conflicts between peers, students, parents, or the community related to SHE were perceived as barriers to effective SHE policy implementation, as divergent opinions led to an overall hostile effect.

At the organizational level (eg, school administration and environment), administrative decisions regarding who is responsible for teaching SHE content and whether adequate training, resources, and instructional materials were available could be perceived as either potential barriers or facilitators of policy implementation.^37^, ^39^ Although SHE training is recommended for educators,^63^ two‐thirds of the participants reported their school administration did not require certification or training to teach SHE.^54^ It was also at this level that participants identified the importance of a school “champion”^65^ to advocate for SHE with district and board leadership.

Having a School Health Advisory Council (SHAC) to help advise staff and administration, or a SBHC with expertise to refer were examples of community‐level influences. When district leaders and parents provided active support of SHE or encouraged concerned parents to seek clarification of course content, those, too, were facilitative community influences. However, some participants viewed community influences as barriers, as when community groups and parents opposed SHE based on political or religious beliefs.^22^, ^39^ When such negative influences were present, schools avoided more sensitive topics or taught them superficially to avoid antagonistic community influences.^22^, ^66^

At the policy level of the SEM, state agencies were identified as potential partners for sharing resources with districts and educators, and as a potential source for training and curricular materials designed to meet policy requirements and standards. However, the lack of evaluation by state agencies also led to the inability to determine whether curricula and teaching methods were effective or congruent with state policies relevant to SHE.

### Limitations

Limitations of the study included use of a convenience sample within a single state and self‐reported data from participants who spoke from personal perceptions. Not all individuals involved in SHE in public secondary schools in the state had the opportunity to participate, and the response rate was 42%. In addition, we did not sample personnel from schools with tribal governance because of the time and travel needed for multiple tribal approvals. Therefore, the generalizability of results is limited. As a descriptive study, there were no a priori hypotheses and no formal statistical comparisons by region, community type, school, or professional role. The analysis of data from unstructured survey questions did not involve specific qualitative analysis techniques. The time needed to complete the phone interview (approximately 30 minutes) could have been a barrier to participation, and we did not collect information on those who declined to participate. Therefore, we could not analyze the extent of self‐selection bias among those who did respond. Although each of the 3 participant groups have important roles in the delivery of SHE, this analysis does not distinguish among them regarding how SHE policy is implemented. The survey tool was not formally validated, and did not delve deeply into issues of language and culture in SHE delivery. The analysis does not speak directly to how SHE is prioritized in the policy environment of public secondary schools, or how requirements relevant to SHE are reconciled with conflicting demands on school staff. Despite these limitations, the analysis is potentially useful to school staff and policymakers as an overall picture of the current delivery of SHE and the levels of influence that shape its delivery.

### Conclusions

Effective and comprehensive delivery of SHE requires clear policies that are understood and supported by those responsible for policy implementation, especially those at the local, school level. Top‐down policy implementation from the state level is rarely able to account for variability in community sensibilities or social, political, and economic challenges faced by those on the front lines of putting policy into practice.^65^, ^67^ State‐level educational directives are often broad, and implementation may take place at many levels.^68^, ^69^ Results of this study demonstrate wide variations in the SHE content taught to students; the time and resources committed to teaching that content; and how, when, and by whom that content is delivered.

School staff and administrators possess unique knowledge of local support and resources for the delivery of SHE and for addressing gaps in policy implementation such as those faced by the participants in this study. This is particularly the case in rural communities.^39^ The knowledge and experience of school personnel are essential for delivering quality SHE that best addresses student needs, while abiding by state policy. How SHE policy is implemented, whether content meets required standards, and the potential impact on adolescent health outcomes will be greatly influenced by how well staff are provided up‐to‐date teaching materials, regular training resources, and organizational support within their communities.^70^

## IMPLICATIONS FOR SCHOOL HEALTH

This study shows multiple levels of influence on the staff that affect the quality and comprehensiveness of SHE. Greater engagement with front‐line personnel is key to reducing barriers to the successful delivery of SHE for public, secondary school students and to ensure support for effective implementation of state‐level policies. Specific implications for school health include:

- Include content related to pedagogy and delivery of SHE in pre‐professional academic preparation and the continuing professional education for both secondary school educators and school nurses.
- Use teaching expertise of teachers and health expertise of nurses to inform content, and evaluate delivery of secondary school SHE curriculum.
- Communicate written state and local policy to all staff and parents indicating SHE standards, the evidence supporting SHE policy, and any opt‐out policy.
- Engage staff responsible for teaching SHE to find out what they need to teach to required policy standards. Explore SHE distance‐learning programs to address prohibitive financial and travel barriers, and engage schools in underserved and under‐resourced areas to share resources, curricula, and access to expertise.
- Consider students' cultural and language needs in preparation of SHE content and resources to fit local cultural context.
- Evaluate state SHE policies in schools and districts using a recognized framework to determine effectiveness, and identify barriers to and facilitators of policy implementation.

### Human Subjects Approval Statement

The study was approved by the University of New Mexico Health Sciences Center Human Research Protections Office. The review determination was exempt, category 2 for surveys/interviews. Approved contact and consent scripts were used, and verbal consent was obtained and documented for participants at the time of the phone interview.

### Conflict of Interest

The authors report no conflict of interest.
